# Disease Activity-Related Sleep Dysfunction in Psoriasis: Insights from a Cross-Sectional Study

**DOI:** 10.3390/jcm15114198

**Published:** 2026-05-29

**Authors:** Damiano Currado, Claudio Conforti, Francesca Trunfio, Annalisa Marino, Marta Vomero, Erika Corberi, Francesca Saracino, Chiara Retrosi, Onorina Berardicurti, Iris Zalaudek, Roberto Giacomelli, Luca Navarini

**Affiliations:** 1 Rheumatology and Clinical Immunology, Department of Medicine, University of Rome Campus Biomedico, School of Medicine, 00128 Rome, Italy; d.currado@policlinicocampus.it (D.C.);; 2 Clinical and Research Section of Rheumatology and Clinical Immunology, Fondazione Policlinico Universitario Campus Bio-Medico, via Alvaro del Portillo 200, 00128 Rome, Italy; 3 Department of Life Science, Health and Health Professions, Link University of Rome, 00165 Rome, Italy; 4 Department of Life, Health and Environmental Sciences, University of L’Aquila, via Vetoio SNC, Ed. Camillo de Meis, 67100 L’Aquila, Italy; 5 IDI-IRCCS, via Monti di Creta 104, 00167 Rome, Italy; 6 Rheumatology, Department Life Sciences, Health and Health Professions, Link Campus University, 00165 Roma, Italy; 7 Department of Dermatology, Maggiore Hospital, University of Trieste, 34127 Trieste, Italy

**Keywords:** psoriasis, sleep quality, PASI

## Abstract

**Background/Objectives:** Psoriasis (PsO) is a chronic immune-mediated disease associated with a substantial multidimensional burden extending beyond skin involvement. Sleep disturbance represents an important yet underexplored domain of patient-reported outcomes. This study aimed to investigate the relationship between PsO disease activity and sleep quality, focusing on specific sleep domains, and to evaluate whether disease severity independently predicts sleep latency and daytime dysfunction. **Methods:** We conducted a cross-sectional study including 136 consecutive patients with PsO from two Italian centers. Disease severity was assessed using the Psoriasis Area and Severity Index (PASI). Sleep quality was evaluated using the Pittsburgh Sleep Quality Index (PSQI), while quality of life was measured by the SF-36. Patients were stratified according to disease severity (PASI < 10 vs. ≥10). Associations between disease activity and sleep domains were analyzed using univariable and multivariable ordinal regression models adjusted for demographic and clinical variables. **Results:** The study population had predominantly low disease activity (median PASI 2). The median global PSQI score was 5, indicating overall borderline sleep impairment. Patients with PASI ≥ 10 showed significantly worse sleep latency (*p* = 0.01) and greater daytime dysfunction (*p* = 0.02), while no significant differences were observed in global PSQI scores. In multivariable analyses, PASI ≥ 10 remained independently associated with increased sleep latency (β = 0.95, 95% CI 0.08–1.82; *p* = 0.032) and daytime dysfunction (β = 2.52, 95% CI 1.31–3.73; *p* < 0.001), after adjustment for confounders. **Conclusions:** Higher PsO disease activity is independently associated with impairment in specific sleep domains, particularly sleep latency and daytime dysfunction, rather than global sleep quality. These findings highlight the importance of assessing domain-specific sleep disturbances and support the integration of sleep outcomes into a holistic, patient-centered approach to PsO management.

## 1. Introduction

Psoriasis (PsO) is a chronic immune-mediated inflammatory disease affecting approximately 2–3% of the general population and is associated with a substantial physical and psychosocial burden [1].

In fact, the disease extends far beyond cutaneous manifestations, exerting a profound impact on patients’ physical, emotional and psychosocial well-being [2]. Although PsO does not typically affect survival, its burden on daily life has been shown to be comparable to that of other major chronic diseases, such as cardiovascular disease and diabetes [3]. Consequently, the assessment of disease impact increasingly relies not only on objective measures of skin involvement, but also on patient-reported outcomes (PROs) reflecting quality of life and functional status [1,4].

Importantly, in patients with psoriatic disease—particularly those with psoriatic arthritis (PsA)—pain and impaired quality of life represent central and often interrelated domains, only partially explained by objective measures of inflammation, thus highlighting the need for a more comprehensive, patient-centered assessment of disease burden [5,6,7,8,9,10,11].

Disease severity in psoriasis is commonly quantified using the Psoriasis Area and Severity Index (PASI), which captures the extent and intensity of skin involvement [12]. However, previous evidence has consistently shown a weak correlation between PASI scores and global measures of quality of life (QoL), suggesting that disease activity and patient-perceived burden represent partially distinct dimensions of the disease [1,13]. This discrepancy highlights the need to better understand which specific aspects of daily functioning are most closely linked to disease severity and therefore potentially modifiable through adequate disease control.

Sleep disturbance represents a particularly relevant, yet underexplored, dimension of disease burden in PsO. Impaired sleep has been associated with fatigue, reduced daytime functioning, mood disturbances and diminished QoL, all of which substantially contribute to patients’ overall perception of disease impact [14,15]. Importantly, sleep is a multidimensional construct that encompasses not only sleep duration and efficiency, but also qualitative aspects such as sleep latency and daytime dysfunction, which may have direct consequences on daily performance and social participation [14,15,16]. Recent population-based evidence has further confirmed a significant association between PsO and sleep disturbance, independent of sleep duration, highlighting the clinical relevance of sleep quality rather than sleep quantity in this population [17].

Several factors may contribute to sleep dysfunction in PsO, including pruritus, pain, psychological distress and systemic inflammation [14,18,19]. However, the relative contribution of disease activity itself to specific domains of sleep impairment remains incompletely understood. Previous studies investigating sleep in PsO have primarily focused on global sleep quality scores or the prevalence of insomnia symptoms, often yielding heterogeneous results [20,21].

Moreover, the relationship between objective disease severity and distinct sleep domains has been variably reported, with some studies suggesting an association between higher PASI scores and poorer sleep quality, while others have failed to demonstrate a clear link [14,20,21]. These inconsistencies may be partly explained by differences in study design, patient populations, and sleep assessment tools.

Notably, few studies have explored whether PsO disease activity is independently associated with specific components of sleep dysfunction—such as sleep latency and daytime dysfunction—beyond the influence of demographic factors and overall health-related quality of life [14,22,23]. A better understanding of this relationship may have important clinical implications, supporting a more holistic, treat-to-target approach in which achieving adequate disease control translates not only into skin improvement, but also into meaningful benefits in daily functioning and wellbeing.

## 2. Objectives

The aim of this study was to investigate the association between PsO disease activity and sleep quality in a real-life cohort of patients with PsO, with a particular focus on individual sleep domains assessed by the Pittsburgh Sleep Quality Index (PSQI). Additionally, we sought to determine whether disease severity independently predicts sleep latency and daytime dysfunction after adjustment for demographic, clinical and quality-of-life variables.

## 3. Materials and Methods

A cross-sectional study was conducted on patients with PsO enrolled in a cohort of psoriatic patients from Trieste and Rome (Italy). Consecutive participants were recruited between January 2021 and July 2021. The study was approved by the Ethics Committee of the University Campus Bio-Medico di Roma and was conducted in accordance with the Declaration of Helsinki and its subsequent amendments.

Inclusion criteria were: age > 18 years, both sexes, and a previous diagnosis of PsO, established by a board-certified dermatologist. Exclusion criteria included a history of any psychiatric disorder according to DSM-V prior to recruitment, history of malignancy, pregnancy, age > 75 years, or inability to provide informed consent.

At enrolment, disease severity was assessed using the PASI. Functional disability and physical function were evaluated using the Health Assessment Questionnaire (HAQ).

Health-related quality of life (QoL) was assessed using the Italian version of the Medical Outcomes Study Short Form-36 (SF-36), which provides two summary measures—the Physical Component Summary (PCS) and the Mental Component Summary (MCS) [24].

Sleep quality was evaluated using the Italian version of the PSQI, a self-administered questionnaire assessing sleep quality and disturbances over a one-month period. The PSQI consists of 19 items grouped into seven components: subjective sleep quality, sleep latency, sleep duration, habitual sleep efficiency, sleep disturbances, use of sleeping medication, and daytime dysfunction. Each component is scored from 0 to 3, with higher scores indicating greater impairment. The sum of the seven components yields a global PSQI score ranging from 0 to 21.

Continuous variables are reported as median and interquartile range (25th–75th percentile), while categorical variables are expressed as percentages. Data distribution was assessed using the Shapiro–Wilk test. The chi-square test was used for the analysis of contingency tables. Comparisons between groups were performed using the Mann–Whitney U test or the Kruskal–Wallis test, with Holm’s correction for pairwise comparisons, as appropriate. Variables associated with sleep disturbance were evaluated using univariable and multivariable ordinal regression models. All statistical analyses were performed using Stata version 14 (StataCorp, College Station, TX, USA). A two-sided *p*-value < 0.05 was considered statistically significant.

## 4. Results

A total of 136 patients with PsO were included in the study. The main demographic, anthropometric and clinical characteristics of the study population are reported in Table 1. All participants were Caucasian (100%), with a predominance of male patients (69.2%) and a median age of 58 years (IQR 49–69).

At enrolment, disease activity was generally low, with a median PASI score of 2 (IQR 1–4.5). PROs indicated minimal functional disability, with a median HAQ score of 0 (IQR 0–0.25). Median values for health-related quality of life were 51.77 (IQR 39.83–55.19) for the Physical Component Summary (PCS) and 49.32 (IQR 41.79–54.01) for the Mental Component Summary (MCS).

The distribution of median values for individual PSQI components was as follows: subjective sleep quality 1.0 (IQR 0.0–2.0), sleep latency 1.0 (IQR 0.0–2.0), sleep duration 1.0 (IQR 1.0–1.0), habitual sleep efficiency 0.0 (IQR 0.0–0.0), sleep disturbances 1.0 (IQR 1.0–2.0), use of sleeping medication 1.0 (IQR 1.0–1.0), and daytime dysfunction 0.0 (IQR 0.0–1.0). The median global PSQI score in the overall PsO population was 5 (IQR 3–8).

To explore the potential impact of disease activity on sleep disturbance, patients were stratified into mild–moderate PsO (PASI < 10) and severe PsO (PASI ≥ 10), as reported in Table 2. A significantly higher proportion of female patients was observed in the PASI < 10 group compared with the PASI ≥ 10 group (*p* = 0.02). Patients with PASI ≥ 10 also exhibited a higher body mass index (*p* = 0.01).

Regarding PROs, patients with PASI ≥ 10 showed significantly worse physical quality of life, as reflected by lower PCS scores (*p* = 0.05). Importantly, severe disease activity was associated with greater impairment in specific sleep domains, with significantly higher sleep latency scores (*p* = 0.01) and greater daytime dysfunction (*p* = 0.02) compared with patients with PASI < 10, while the global PSQI score did not differ significantly between groups.

To further investigate the relationship between disease activity and sleep domains, univariable and multivariable ordinal regression analyses were performed for sleep latency and daytime dysfunction (Table 3 and Table 4). In multivariable models adjusted for age, sex, disease duration, body mass index, PCS and MCS, PASI ≥10 remained independently associated with increased sleep latency (β = 0.95, 95% CI 0.08–1.82; *p* = 0.032) and with greater daytime dysfunction (β = 2.52, 95% CI 1.31–3.73; *p* < 0.001).

## 5. Discussion

In this cross-sectional observational study, we investigated the relationship between PsO disease activity and sleep quality in a real-life cohort of patients with PsO, with a specific focus on individual sleep domains. Our findings demonstrate that higher disease activity, as assessed by PASI, is associated with poorer sleep quality, particularly affecting sleep latency and daytime dysfunction. Importantly, these associations remained significant after adjustment for demographic factors and health-related quality of life, suggesting an independent effect of disease severity on specific components of sleep dysfunction.

While the global PSQI score did not differ significantly between patients with mild–moderate and severe disease, individuals with PASI ≥ 10 exhibited greater difficulty initiating sleep and more pronounced impairment in daytime functioning. These results highlight that the impact of PsO on sleep is not adequately captured by global sleep quality measures alone, but rather emerges at the level of specific, clinically meaningful sleep domains. Sleep latency and daytime dysfunction may represent sensitive indicators of disease burden, as they directly influence daily performance, social participation and overall wellbeing. Our findings align with previous recommendations calling for a more granular assessment of sleep disturbance in PsO, as sleep disruption may have relevant consequences on immune regulation, sympathetic nervous system activity and overall disease burden [15,22]. Moreover, our findings help clarify previous heterogeneous observations regarding the relationship between PsO severity and sleep disturbance [21,22].

Earlier studies have variably reported associations between PASI and sleep quality, often relying on global sleep scores or insomnia prevalence and yielding inconsistent results [20,21,22]. Importantly, while several previous studies have described associations between psoriasis severity and sleep impairment, most relied on global sleep scores or unadjusted analyses, limiting their ability to capture the independent contribution of disease activity to specific sleep domains [22,23]. By focusing on individual PSQI components and applying multivariable models, our study provides evidence that disease activity is specifically linked to qualitative aspects of sleep, rather than to sleep duration or efficiency per se. This domain-specific effect may explain why prior studies failed to demonstrate a consistent association when using composite sleep measures. From a clinical perspective, these results have relevant therapeutic implications. The observation that patients with PASI ≥ 10 experience greater impairment in sleep latency and daytime functioning suggests that achieving better disease control—particularly maintaining PASI below clinically relevant thresholds—may translate into meaningful improvements in sleep and daily functioning.

Although causal inferences cannot be drawn due to the cross-sectional design, our findings support the hypothesis that disease activity may act as a clinically relevant contributor to sleep dysfunction, warranting further longitudinal investigation. In this context, sleep outcomes may represent an additional, patient-centred target within a holistic, treat-to-target approach to psoriasis management, complementing traditional measures of skin involvement.

Some limitations of this study should be acknowledged. The relatively limited sample size and the cross-sectional design preclude causal inferences and may not fully capture the heterogeneity of PsO. In addition, the retrospective definition of disease remission may have introduced some degree of misclassification.

Nevertheless, the use of validated PRO measures and multivariable analyses strengthens the robustness of our findings and supports their clinical relevance.

## 6. Conclusions

Taken together, our findings indicate that PsO disease activity has clinically meaningful consequences that extend beyond skin involvement and are reflected in specific domains of sleep dysfunction. Higher PASI scores were independently associated with impaired sleep latency and greater daytime dysfunction, highlighting that disease severity influences not only visible manifestations but also patients’ daily functioning and wellbeing. These results support the integration of sleep-related outcomes into a comprehensive assessment of psoriasis, bridging traditional dermatological measures with patient-reported domains that capture the lived experience of the disease. Achieving and maintaining low PsO disease activity may therefore confer benefits that extend beyond cutaneous improvement, translating into better sleep-related functioning and enhanced quality of life for patients with PsO.

## Figures and Tables

**Table 1:** Demographic, clinical and sleep characteristics of patients with psoriasis.

Variables	PsO Participants (n = 136)
Age, years	58 (49–69)
Female	42 (30.8)
Duration median, months	204 (48–408)
BMI	25.6 (23.3–28.2)
PASI	2 (1–4.5)
HAQ	0 (0–0.25)
PCS	51.77 (39.83–55.19)
MCS	49.32 (41.79–54.01)
Sleep Quality	1 (0–2)
Sleep Latency	1 (0–2)
Sleep Duration	1 (1–1)
Sleep Efficiency	0 (0–0)
Sleep Disturbance	1 (1–2)
Sleep Medications	1 (1–1)
Daytime dysfunction	0 (0–1)
Sleep Global Score	5 (3–8)

Data are reported as median (interquartile range) or number (percentage), as appropriate. PsO: psoriasis; PASI: Psoriasis Area and Severity Index; BMI: body mass index; HAQ: Health Assessment Questionnaire; PCS: Physical Component Summary; MCS: Mental Component Summary.

**Table 2:** Demographic, clinical and sleep characteristics of patients with psoriasis stratified by disease severity.

Variables	PsO Participants with PASI < 10 (n = 112)	PsO Participants with PASI ≥ 10 n = (24)	*p*-Value
Age, years	58 (49–70)	56.5 (52–66.5)	0.83
Female	39 (34.82)	3 (12.5)	**0.02**
Duration median, months	168 (48–360)	300 (120–426)	0.19
BMI	25.2 (23.3–27.7)	28.9 (23.9–29.8)	**0.01**
PASI	1 (0–2)	16.5 (12–20.5)	**<0.0001**
HAQ	0 (0–0.32)	0 (0–0.05)	0.09
PCS	49.82 (39.37–54.84)	52.28 (49.25–58.52)	0.05
MCS	49.87 (42.40–54.32)	46.53 (33.56–50.39)	0.14
Sleep Quality	1 (0–2)	0 (0–1)	0.09
Sleep Latency	1 (0–2)	1 (2–3)	**0.01**
Sleep Duration	1 (1–1)	1 (1–1)	0.5
Sleep Efficiency	0 (0–0)	0 (0–0)	0.8
Sleep Disturbance	1 (1–2)	1 (1–2)	0.3
Sleep Medications	1 (1–1)	1 (1–1.5)	0.1
Daytime dysfunction	0 (0–1)	0.5 (0–1.5)	**0.02**
Sleep Global Score	5 (3–8)	6 (5–8.5)	0.08

Data are reported as median (interquartile range) or number (percentage), as appropriate. PsO: psoriasis; PASI: Psoriasis Area and Severity Index; BMI: body mass index; HAQ: Health Assessment Questionnaire; PCS: Physical Component Summary; MCS: Mental Component Summary; Bold *p*-values indicate statistical significance (*p* < 0.05).

**Table 3:** Univariable and multivariable regression, sleep latency as dependent variable.

Independent Variables	Univariable				Multivariable			
	Coef.	95%CI		*p*−Value	Coef.	95%CI		*p*-Value
Age	0.0402669	0.0163419	0.064192	**0.001**	0.0452161	0.0150193	0.0754129	**0.003**
Female	0.1938979	−0.5172723	0.9050681	0.593	0.1316692	−0.7821005	1.045439	0.778
Disease duration	0.0027659	0.0010098	0.004522	**0.002**	0.0011039	−0.0009329	0.0031408	0.288
BMI	0.0728147	0.0180918	0.1275375	**0.009**	0.0027743	−0.0761512	0.0816997	0.945
PASI	0.0780125	0.032415	0.12361	**0.001**				
HAQ	2.590877	1.549321	3.632434	**<0.0001**				
PCS	−0.05563	−0.0852671	−0.0259929	**<0.0001**	−0.0404982	−0.0744404	−0.0065559	**0.019**
MCS	−0.066456	−0.0980817	−0.0348302	**<0.0001**	−0.0651624	−0.1060589	−0.0242659	**0.002**
PASI ≥ 10	0.8484127	0.1178261	1.578999	**0.023**	0.9512953	0.0813892	1.821201	**0.032**

Values are expressed as regression coefficients (Coef.) with 95% confidence intervals (95% CI) and *p*-values. Multivariable models were adjusted for age, sex, disease duration, body mass index (BMI), Health Assessment Questionnaire (HAQ), Physical Component Summary (PCS) and Mental Component Summary (MCS). Bold *p*-values indicate statistical significance (*p* < 0.05).

**Table 4:** Univariable and multivariable regression, daytime dysfunction as dependent variable.

Independent Variables	Univariable				Multivariable			
	Coef.	95%CI		*p*-Value	Coef.	95%CI		*p*-Value
Age	0.0421105	0.0135097	0.0707113	**0.004**	0.1234882	0.0705747	0.1764017	**<0.0001**
Female	−0.2512724	−1.006311	0.5037665	0.514	1.844594	0.5457594	3.143429	**0.005**
Disease duration	0.0037982	0.0018134	0.005783	**<0.0001**	0.002387	−0.0003834	0.0051573	0.09
BMI	−0.0915222	−0.1868566	0.0038122	0.06	−0.706076	−0.9864165	−0.4257355	**<0.0001**
PASI	0.0549466	0.0012013	0.1086919	**0.045**				
HAQ	0.7457392	−0.1291441	1.620622	0.09				
PCS	−0.0448934	−0.0756646	−0.0141222	**0.004**	−0.0415429	−0.0844072	0.0013213	0.05
MCS	−0.0402894	−0.0712073	−0.0093715	**0.01**	−0.1364957	−0.1916262	−0.0813652	**<0.0001**
PASI ≥ 10	0.9546421	0.0975454	1.811739	**0.029**	2.519654	1.307997	3.731312	**<0.0001**

Values are expressed as regression coefficients (Coef.) with 95% confidence intervals (95% CI) and *p*-values. Multivariable analyses were adjusted for age, sex, disease duration, body mass index (BMI), Health Assessment Questionnaire (HAQ), Physical Component Summary (PCS) and Mental Component Summary (MCS). Bold *p*-values indicate statistical significance (*p* < 0.05).

## Data Availability

The data presented in this study are available on request from the corresponding author.

## Abbreviations

The following abbreviations are used in this manuscript:

PsO	Psoriasis
PsA	Psoriatic Arthritis
PASI	Psoriasis Area and Severity Index
PSQI	Pittsburgh Sleep Quality Index
SF-36	Short Form-36 Health Survey
QoL	Quality of Life
PROs	Patient-Reported Outcomes
HAQ	Health Assessment Questionnaire
PCS	Physical Component Summary
MCS	Mental Component Summary
CI	Confidence Interval
DSM-V	Diagnostic and Statistical Manual of Mental Disorders, Fifth Edition
IQR	Interquartile Range
